# Gold Nanorods as Saturable Absorber for Harmonic Soliton Molecules Generation

**DOI:** 10.3389/fchem.2019.00715

**Published:** 2019-10-24

**Authors:** Yiqing Shu, Penglai Guo, Xiaohui Li, Guian Li, Peng Wang, Ge Shen, Jianqing Li

**Affiliations:** ^1^School of Physics and Information Technology, Shaanxi Normal University, Xi'an, China; ^2^School of Electronic Information Technology, Macau University of Science and Technology, Macau, China

**Keywords:** gold nanorods, non-linear optical properties, ultrafast photonics, mode-locked fiber laser, harmonic soliton molecules

## Abstract

Gold nanorods (GNRs) has been investigated in the field of chemistry, optoelectronics, and medicine for their tenability, compatibility, electromagnetics, and excellent photonics properties. Especially, GNRs, used to generate ultrashort pulse, have been studied recently. However, multiple pulses evolution based on GNRs needs to be further explored. In this article, GNRs are synthesized by seed-mediated growth method, characterized systematically and been chosen as saturable absorber (SA) to apply in ultrafast photonics. The GNRs SA presents a saturable intensity of 266 MW/cm^2^, modulation depth of 0.6%, and non-saturable loss of 51%. Furthermore, a passively mode-locked erbium-doped fiber laser based on GNRs SA with femtosecond pulse is demonstrated. Thanks to the excellent properties of GNRs, by adjusting the cavity polarization direction with the proposed GNRs SA, soliton molecules operation with spectrum modulation period of 3.3 nm and pulse modulation interval of 2.238 ps is directly obtained. For the most important, 9th-order harmonic soliton molecules have been generated in the laser cavity for the first time. It is demonstrated that GNRs can be a novel type of non-linear optical (NLO) device and have potential applications in the field of ultrafast photonics.

## Introduction

Many researchers have been attracted by noble metal nano-materials, such as gold, silver, due to the strong non-linear optical (NLO) effects which caused by their surface plasmon resonances (SPR) (Oh et al., 2011; Tsutsui et al., 2011; Huang et al., 2014; Komarov et al., 2015; Fan and Zhang, 2016; Yang et al., 2018). These effects enable noble metal nano-materials get many potential applications in synthesize NLO devices, such as optical detectors, optical sensors, and optical absorbers (Jain et al., 2007; Zijlstra et al., 2009). Among these noble metal nano-materials, gold nanorods (GNRs) have two SPR peaks, one is transverse SPR (TSPR) and the other is longitudinal SPR (LSPR). The LSPR is particularly sensitive to the aspect ratio of the GNRs and could be flexibly tuned through a broad spectral ranging from the visible to the near-infrared regime (Gou and Murphy, 2005; Ming et al., 2009; Kang et al., 2013; Fang et al., 2014; Lopez-Lozano et al., 2014; Burrows et al., 2016; He et al., 2016). This indicates that GNRs could be used in diverse wavebands when they are needed. In recent years, mode-locked and Q-switched fiber lasers using GNRs as SA have been demonstrated at different wavebands (1, 1.56, and 2 μm) (Kang et al., 2013, 2015; Wang X. et al., 2016; Chen et al., 2018).

Up to now, enormous experimental data and simulation results show that optical fiber lasers can produce many categories of pluses, such as conventional solitons (CSs), dissipative solitons, stretched pulse, self-similar pulse (Peng et al., 2013; Li et al., 2016; Wang Y. et al., 2016; Chai et al., 2018; Zhang et al., 2018; Wang et al., 2019). At the state of multi-soliton, harmonic mode locking (HML) would be formed when the solitons repel each other in a long range and uniformly fill in the whole laser cavity (Yang et al., 2007; Wang Z. et al., 2015; Fu et al., 2019). In addition, another kinds of multiple pulse called bound solitons (BSs) or soliton molecules would be formed as well, when the solitons repel each other in a short range and attract each other in a long range then transmit simultaneously (Komarov et al., 2008; Wang Y. et al., 2015).

Generally, fiber laser would work in one of the multi-soliton states. Nevertheless, coexistence between different kinds of multi-soliton states could also be generated in a passively mode-locked fiber lasers by precisely adjusting and optimizing the cavity parameters (Zhao et al., 2009; Luo et al., 2018; Zhang et al., 2019). Recently, researchers have reported that the two kinds of pulses, harmonic soliton molecules and rectangular noise-like pulses, can be coexisted in a fiber laser (Huang et al., 2016). In addition, different real saturable absorbers (SAs) can also be excellent composed for the generation of multiple pulses in the fiber laser cavity (Martinez et al., 2017; Lai et al., 2018). Other researchers reported that bound soliton and harmonic mode-locking soliton could be coexisted in an ultrafast fiber laser by MoS_2_ SA for a photonic device (Liu et al., 2018). GNRs as a promising SA need to be further investigated in aspect of generating special kinds of multiple pulses in the fiber laser cavities (Lu et al., 2018).

Although some studies have reported silica-encased GNRs can also be used as SA to produce ultrashort pulses, the preparation process of SA is more complex and only traditional solitons are obtained in fiber laser cavity (Wang X. et al., 2016). In this article, GNRs are successfully synthesized by seed-mediated growth method and characterized by scanning electron microscope (SEM), transmission electron microscope (TEM), and absorption spectra in the visible and near-infrared regions (Johnson et al., 2002; Perezjuste et al., 2005; Chow, 2017). Then, harmonic mode-locking of soliton molecules in EDF laser based on GNRs SA is studied. Depositing the GNRs dispersion on a taper fiber, whose waist diameter and length of 15 μm and 1.5 mm, respectively, to fabricate SA. Using the GNRs SA to obtain ultrashort pulse in infrared region. The GNRs SA has been studied with a modulation depth of 0.6% and non-saturable loss of 51%. By adjusting the laser's polarization direction, 740.6 fs soliton molecules are directly obtained from the Er-doped fiber laser based on GNRs SA. Increasing the pump power, 9th-order harmonic soliton molecules are generated in the laser cavity for the first time. It's much faster than many works before (Kang et al., 2013). The SA exhibits excellent stability, after a month, a steady pulse can be obtained as before. This work further confirms that the GNRs are promising high-performance SA with many potential applications in many fields such as optical fiber communications, optical logic signal processing, and even materials processing, etc.

## Materials and Synthesis of GNRs

### Materials

Chloroauric acid (HAuCl_4_), hexadecyltrimethyl ammonium bromide (CTAB), sodium borohydride (NaBH_4_), silver nitrate (AgNO_3_), hydrochloric acid (HCl), ascorbic acid (AA). All the glass wares are cleaned with deionized water by ultrasonic cleaning machines prior to the experiments.

### Synthesis of GNRs

GNRs are synthesized by using the seed-mediated growth method reported previously. Briefly, HAuCl_4_ (marked solution B) is added to 8 mL CTAB (marked solution A) and mixed in a magnetic stirrer at 35°C, NaBH_4_ is added to the mixture with continuous stirring for 2 min. The color of the solution immediately changes from luminous yellow to dark brown. This change suggests that gold nanoparticles are successfully synthesized. The formed gold nanoparticles dispersion is sat in an incubator for 2 h to grow into seed solution and the seed solution are stable for a couple of weeks.

**Figure 1:**
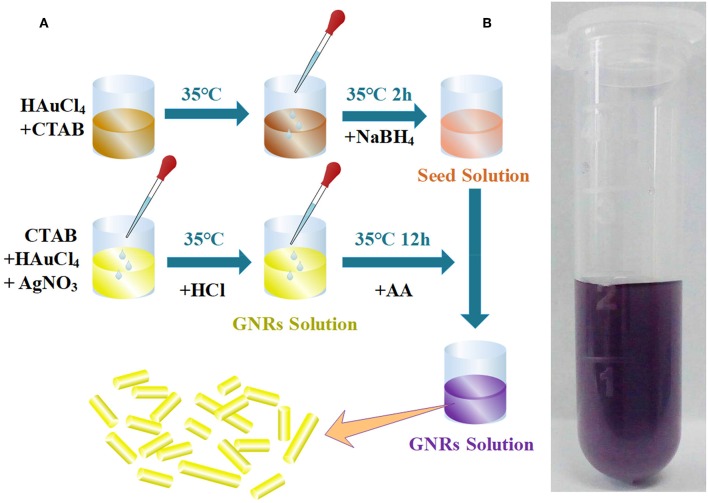
**(A)** Preparation process of GNRs. **(B)** Photograph of GNRs water solution.

AgNO_3_ (marked solution c) and HAuCl_4_ (marked solution b) are added, respectively, to 20 mL CTAB (marked solution a), then added HCl to the solution (a) and mixed on a magnetic stirrer at 35°C (marked solution 1), AA is added to growth solution. Adding seed solution to growth solution and setting the mixture in an incubator for 12 h. Using deionized water to wash resulting solution repeatedly and centrifuging the resulting solution at 8,000 revolutions per min for 10 min once a time. Keeping the resulting solution in cold storage for further characterization. Figure 1A shows the preparation process of GNRs. Figure 1B shows the photograph of GNRs solution.

## Characterization of Gold Nanorods (GNRs)

**Figure 2:**
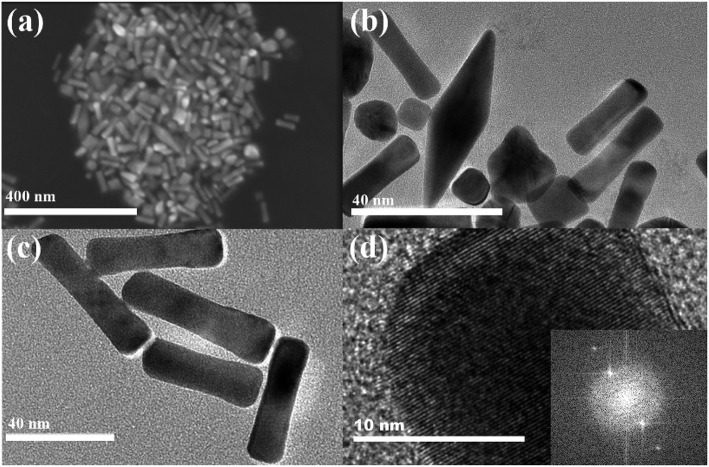
**(a)** SEM image of gold nanomaterials. **(b,c)** TEM images of GNRs with 40 nm scale. **(d)** HR-TEM image of GNR with 10 nm scale, the inset shows the power spectrum of the image.

The SEM (the scale bar is 400 nm) and TEM (the scale bar is 40 nm) images of samples are shown in Figures 2a–c to characterize the obtained GNRs. Obviously, rod-shaped structure of samples are in the majority. By measuring we get the aspect ratio of GNRs about 2.5–8.5. It is interesting to note that seed particles also produce a significant amount of small rods and other secondary shapes, such as cone-shaped. In Figure 2d shows TEM image (HR-TEM) of GNRs. We can clearly observe a crystal lattice of GNRs in this image. The inset of Figure 2d shows the corresponding selective electron diffraction pattern. The GNRs prepared by seed growth method are single crystal structure, which is also indicated by the high resolution TEM of GNRs and the corresponding selective electron diffraction pattern.

**Figure 3:**
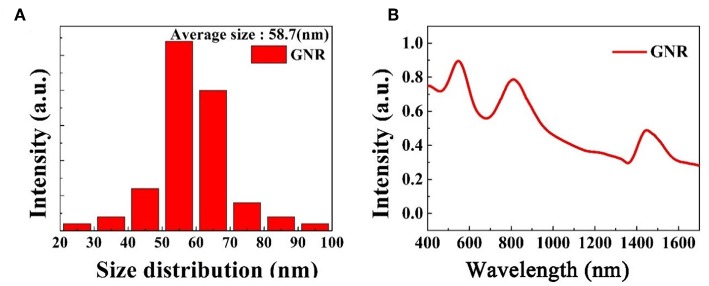
**(A)** Size distribution of GNRs. **(B)** Absorption spectra of GNRs water solution.

Figure 3A shows the size distribution of the gold nanomaterials. The average diameters of these GNRs are 58.7 nm. The visible-near infrared (VIS-NIR) absorption is shown in Figure 3B. The samples have three higher absorption bands peaked at 0.5, 0.8, 1.5 μm, respectively. The absorption band peaked at 0.5 μm is caused by the TSPR with the diameter of GNRs is ~14 nm. The second band peaked at 0.8 μm is caused by the small GNRs and other secondary shapes. The band we are interested in 1.5 μm caused by the LSPR of long GNRs, which provides a usability of our GNRs sample in ultrafast laser as a SA.

**Figure 4:**
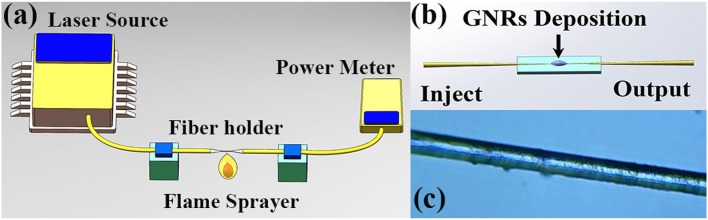
**(a)** Experimental setup for fabricating a taper fiber. **(b)** Schematic diagram for deposition of GNRs material on taper fiber by injecting laser. **(c)** Image of the microscopical taper fiber-based GNRs SA.

The fabrication processes of GNRs SA are described in Figure 4. After mechanical exfoliation, the bare fiber of single mode fiber (SMF) is burned and stretched, only in this way can we prepare a segment of taper fiber with waist diameter of 15 μm and length of 1.5 mm, this process is shown in Figure 4a. Figure 4b exhibits a model for preparing the GNRs SA by means of optical induced deposition. Injecting laser in the end of microfiber when dropping GNRs dispersion onto the surface of taper fiber. Figure 4c depicted a taper fiber reality image observed under a microscope and the black part attached to the surface of taper fiber is GNRs.

**Figure 5:**
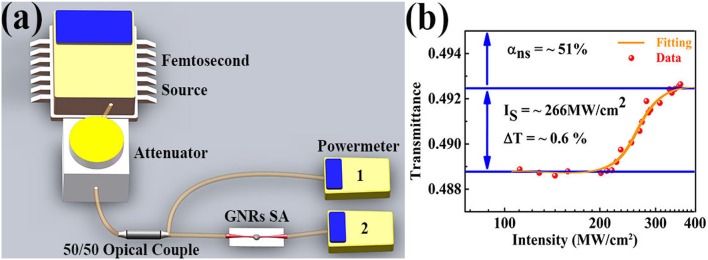
**(a)** Measurement schematic of non-linear saturable absorption. **(b)** The saturable absorption curvey of gold nanomaterials SA.

In order to further investigate the characteristics of GNRs, the non-linear absorption is measured by using balanced twin detector measurement technology. Figure 5a describes the measurement method of saturable absorption property about the GNRs. In this experiment, the pump source, a standard femtosecond pulse laser source whose center wavelength of 1563.3 nm, repetition rate of 21.4 MHz and pulse duration of 584 fs is divided into two parts by a 50/50 optical coupler after passing through attenuator which can be adjust the output power of standard laser source. One accesses power meter 1 directly, the other injects the GNRs SA before enters the power meter 2. The optical non-linear transmittance result is exhibited in Figure 5b. As shown here, the saturable intensity is 266 MW/cm^2^, the modulation depth is ~0.6%, and the non-saturable loss of 51%. Based on these experimental results, we apply GNRs as a SA in fiber laser for implementing mode-locking.

## Laser Performance and Discussions

**Figure 6:**
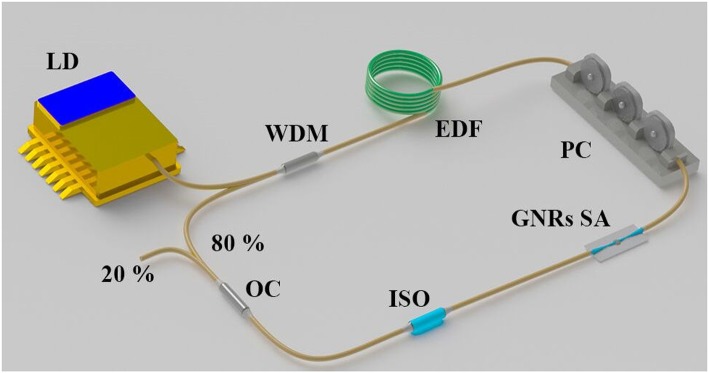
Schematic diagram of the fiber laser cavity.

To check the laser performance of GNRs SA, it is incorporated into a laser cavity to generate ultrashort pulse. The schematic of the passively mode-locked EDF ring laser is shown in Figure 6. A 0.5 m-long EDF offering a laser gain is pumped by 976 nm laser diode (LD) connecting a 980/1,550 nm wavelength division multiplexer (WDM). The polarization controller (PC) are utilized to control the light polarization state for achieving mode-locking and optimizing the laser operation. A polarization-independent isolator (PI-ISO) is used to guarantee the unidirectional operation. The GNRs SA plays an essential role in this fiber laser cavity. A 20/80 fiber optical coupler (OC), the 20% is exported to detect intracavity laser performance and the other 80% turn back laser cavity to oscillate, is located in the end of cavity. The total cavity length is around ~17 m corresponding to a fundamental repetition rate of 11.3 MHz. The spectrum and pulse train are monitored by an optical spectrum analyzer (Anritsu MS9710C), a real-time oscilloscope with resolution of 1 GHz (Rigon DS6104) and a photodetector with resolution of 2 GHz (Thorlabs DET01CFC). The radio frequency (RF) spectrum of the mode-locked operation is recorded by a radio frequency spectrum analyzer (Rohde & Schwarz FSC6). Moreover, the pulse duration and modulation interval are measured by an autocorrelator (Femtochrome FR-103XL).

**Figure 7:**
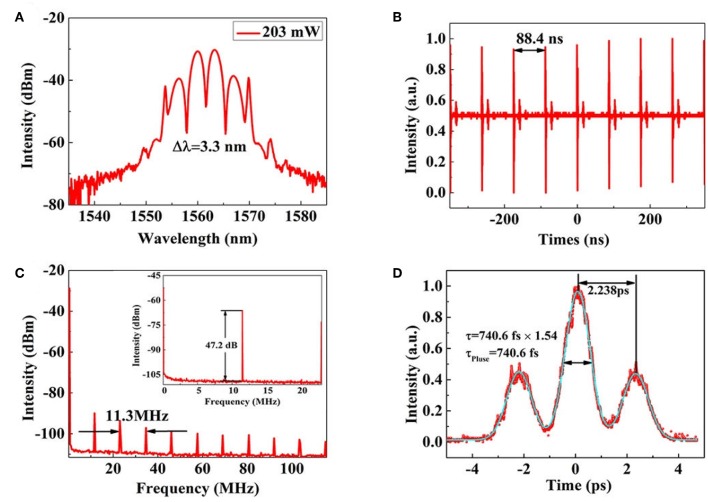
**(A)** The typical soliton molecule spectrum obtained at the pump power of 203 mW. **(B)** The corresponding soliton molecule pulse-sequence. **(C)** RF spectrum around the fundamental repetition rate; **(D)** autocorrelation trace of partially coherent pulse generation.

Then simply increasing the pump power to 153 mW, the self-started soliton molecule mode-locking operation occurs. For further exploring the mode-locking operation, the pump power is increased to 314 mW. Figure 7A shows the typical soliton molecule spectrum obtains at the pump power of 203 mW. Here, spectrum modulation period of soliton molecule is 3.3 nm. Figure 7B presents the corresponding mode-locked pulse-train and we realize the pulse period is 88.4 ns. To verify the laser stability, we measure the radio frequency (RF) spectrum of the mode locking operation with a resolution bandwidth of 30 kHz and video bandwidth of 100 Hz. As presented in Figure 7C, the fundamental frequency peak locates at 11.3 MHz determined by the cavity length of ~17 m, corresponding to the fundamental cavity repetition rate. The signal-to-noise ratio (SNR) is ~47.2 dB, indicating the high stability of mode-locking operation. Moreover, the inset of Figure 7C shows RF spectrum measured under the 22.6 MHz span. Then, by using AC, the soliton modulation period and duration of the mode-locked pulse are identified. As described in Figure 7D, the soliton molecule delivers the pulse-train with 740.6 fs duration and 2.238 ps soliton pulse modulation interval if the fit of the sech^2^ pulse shape is assumed. There is a formula to describe the relationship between spectrum modulation period (Δλ) and pulse modulation interval (ΔT) of soliton molecule:

(1) $$\begin{array}{r} \Delta \text{T}=|\lambda_{0}{|}^{2}/\left(c\cdot \Delta \lambda \right) \end{array}$$

Here, λ_0_ and c mean center wavelength of mode-locked laser operation and speed of light. Their values are 1,560 nm and 3 × 10^8^ m/s, respectively. Through theoretical calculation, our experimental results are consistent with the theoretical predictions.

**Figure 8:**
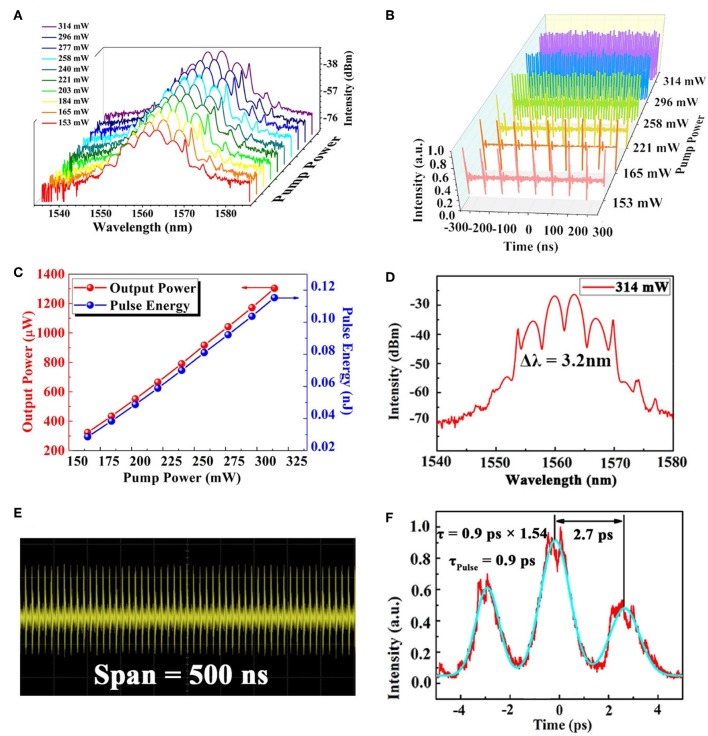
**(A)** Evolution of optical spectrum with the pump power from 153 to 314 mW. **(B)** Pulse sequence evolution with pump power. **(C)** Output power of soliton molecule as a function of pump power. **(D)** Optical spectrum with 314 mW of pump power. **(E)** Pulse sequence of higher order harmonic soliton molecule under the pump power of 314 mW. **(F)** Autocorrelation trace of higher order harmonic soliton molecule under the pump power of 314 mW.

After increasing the pump power gradually to the maximum of laser diode, fiber laser is still working in the mode-locked state. Moreover, the soliton molecule could maintain stable operation well at the maximum pump power of 350 mW, which means the optical damage threshold of GNRs SA is considerable high. Figures 8A,B show the spectrum and sequence evolution of soliton molecule with pump power from 153 to 314 mW. Figure 8C depicts the variation of the soliton molecule operation output power when we increasing the pump power. More importantly, the 9th-order harmonics soliton molecule is obtained during the process of increasing pump power. Optical spectrum, pulse sequence and autocorrelation trace of 9th order harmonics soliton molecule under 314 mW of pump power are exhibited in Figures 8D–F, respectively. We can discover that 9th soliton molecule has the 3.2 nm spectrum modulation period, 101.7 MHz fundamental frequency and 0.9 ps pulse duration with 2.7 ps soliton pulse modulation interval. In the experiment, we also check the significance of GNRs SA in fiber laser. GNRs SA is removed from the laser cavity. As a result, there is no soliton molecule operation even if the PC is rotated in a large range. The results demonstrate that the ultrafast properties of GNRs are accountable for the generation of the fiber laser.

As we know, not only the soliton molecules and harmonic mode-locking solution but many other kinds of pluses could also be generated in a fiber laser, such as soliton rain and noise-like pluses. Nevertheless, in our experiments, only two multi-soliton states are observed. Thanks to the high optical damage threshold of GNRs we would do more research on composite mode-locked operations based on GNRs SA and investigate the coexistence of multi-soliton dynamics.

## Conclusion

In conclusion, GNRs are prepared by seed-mediated growth method and characterized by the technology of SEM, TEM, VIS-NIR absorption which noticed the micro-structure and the absorption of GNRs. By measuring the NLO response of GNRs, the obvious saturable absorption effect is identified which shows a modulation depth of 0.6%. And then, GNRs are used as a high-performance SA in a fiber laser for generating soliton molecule mode-locked pulse. By the GNRs SA, the 740.6 fs stable soliton molecule mode-locked pulse easily produced from the fiber laser. And 9th-order harmonics mode-locked soliton molecules obtained in the laser cavity for the first time. It turns out that the GNRs SA could be a really wonderful NLO material for preparation of saturable absorption device for ultrafast lasers. And has the great potential in many applications.

## Data Availability Statement

All datasets generated for this study are included in the article/supplementary files.

## Author Contributions

YS do experiment and write paper. XL providing guiding ideology and experimental equipment. PG process data and draw experimental images. GL provide materials needed for the experiment. PW participate in the preparation of gold nanorods. GS purchase reagents for the experiment. JL modify the article.

### Conflict of Interest

The authors declare that the research was conducted in the absence of any commercial or financial relationships that could be construed as a potential conflict of interest.
